# Fabrication of Wireless Micro Pressure Sensor Using the CMOS Process

**DOI:** 10.3390/s91108748

**Published:** 2009-10-30

**Authors:** Ching-Liang Dai, Po-Wei Lu, Chyan-Chyi Wu, Chienliu Chang

**Affiliations:** 1 Department of Mechanical Engineering, National Chung Hsing University, Taichung, 402 Taiwan; E-Mail: player0912@hotmail.com; 2 Department of Mechanical and Electro-Mechanical Engineering, Tamkang University, Tamsui, 251 Taiwan; E-Mail: ccwu@mail.tku.edu.tw; 3 Corporate R&D Headquarters, Canon Inc., Tokyo 146-8501, Japan; E-Mail: clchang6@ntu.edu.tw

**Keywords:** wireless micro pressure sensor, CMOS-MEMS, oscillators

## Abstract

In this study, we fabricated a wireless micro FET (field effect transistor) pressure sensor based on the commercial CMOS (complementary metal oxide semiconductor) process and a post-process. The wireless micro pressure sensor is composed of a FET pressure sensor, an oscillator, an amplifier and an antenna. The oscillator is adopted to generate an ac signal, and the amplifier is used to amplify the sensing signal of the pressure sensor. The antenna is utilized to transmit the output voltage of the pressure sensor to a receiver. The pressure sensor is constructed by 16 sensing cells in parallel. Each sensing cell contains an MOS (metal oxide semiconductor) and a suspended membrane, which the gate of the MOS is the suspended membrane. The post-process employs etchants to etch the sacrificial layers in the pressure sensor for releasing the suspended membranes, and a LPCVD (low pressure chemical vapor deposition) parylene is adopted to seal the etch holes in the pressure. Experimental results show that the pressure sensor has a sensitivity of 0.08 mV/kPa in the pressure range of 0–500 kPa and a wireless transmission distance of 10 cm.

## 1. Introduction

Micro pressure sensors, which are important components, can be used in the biomedical and various other industries [1,2]. Microelectromechanical system (MEMS) technology has recently been applied to manufacture micro pressure sensors. The advantages of micro pressure sensors fabricated by MEMS technology include high performance, small size, low cost and easy mass-production. Several micro FET pressure sensors [3-7] have been manufactured by using MEMS technology. For instance, a FET pressure sensor, reported by Hynes *et al.* [3], was fabricated using a surface micromachining process, in which a polysilicon diaphragm and a sacrificial oxide layer were deposited on the pressure sensing area, and then the sacrificial oxide was removed from beneath the polysilicon diaphragm using HF solution. LPCVD oxide film was utilized to seal the cavity of the sensor. The sensitivity of the FET pressure sensor was 1.3 μA/psi. Svensson *et al.* [4] employed a surface micromachining process to manufacture a FET pressure sensor. The pressure sensor was an MOS transistor where the device gate was the membrane, and the source and the drain regions were the two silicon diffusions under the membrane. The FET pressure sensor had a sensitivity of 0.1 mA/bar. Lysko *et al.* [5] presented a FET pressure sensor also fabricated by a surface micromachining technique, and consisting of gate, source, drain and substrate terminals. The gate was the polysilicon membrane doped with arsenic, and a cavity was located in between the polysilicon membrane and the silicon substrate. Dai *et al.* [6] proposed a FET pressure sensor with the readout circuit on a chip manufactured by the commercial CMOS process and a post-process. The readout circuit was utilized to convert the current variation of the pressure sensor into the voltage output. The pressure sensor was composed of several sensing cells in parallel, and each sensing cell consisted of a suspended membrane and an NMOS. The suspended membrane was the movable gate of the NMOS. The post-process was the use of wet etching to remove the sacrificial layers, and to obtain the suspended membrane and the etch holes in the pressure sensor were sealed by LPCVD parylene. The sensitivity of the pressure sensor was about 0.032 mV/kPa. The FET pressure sensor integrated circuits on a chip had the advantages of low packaging cost and small chip area. These FET pressure sensors proposed by Hynes *et al.* [3], Svensson *et al.* [4], Lysko *et al.* [5] and Dai *et al.* [6], did not have integrated wireless circuits on-chip. If the FET pressure sensors could integrate with wireless circuits as wireless pressure sensors they would have more applications, so in this work we have developed a wireless FET pressure sensor based on the commercial CMOS process.

The technique that uses the commercial CMOS process to fabricate MEMS devices is called CMOS-MEMS [7-12]. The advantages of CMOS-MEMS micro pressure sensors include low cost per unit area, compatible with integrated circuits and mass-production utilizing semiconductor foundries. In this study, we employ the CMOS-MEMS technique to manufacture an FET pressure sensor integrated with wireless transmission circuits on a chip. The wireless transmission circuits consist of an oscillator, an amplifier and an antenna. The oscillator generates an ac signal providing to the gate of the FET pressure sensor. The amplifier is utilized to amplify the sensing signal of the pressure sensor, and the antenna is used to transmit the output voltage of the pressure sensor to a receiver. In order to release the suspended membranes, the sacrificial layers in the pressure sensor are removed by the post-process of wet etching. Experimental results show that the FET pressure sensor has a sensitivity of 0.08 mV/kPa in the pressure range of 0-500 kPa and a wireless transmission distance of 10 cm.

## 2. Structure of the Pressure Sensor

**Figure 1.**
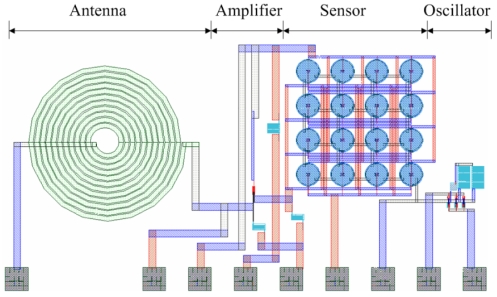
Layout of the wirless pressure sensor.

Figure 1 shows the layout of the wireless pressure sensor consisting of a FET pressure sensor, an oscillator, an amplifier and an antenna. The oscillator is utilized to produce an ac signal, and the amplifier is adopted to amplify the sensing signal of the pressure sensor. The antenna is employed to transmit the output voltage of the pressure sensor to a receiver. The pressure sensor contains 16 sensing cells in parallel. All sensing cells have the same dimensions, and the diameter of each sensing cell is 100 μm.

**Figure 2.**
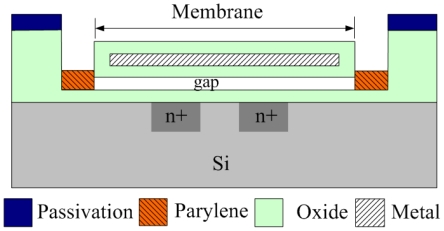
Schematic cross-sectional view of a sensing cell.

Figure 2 illustrates the schematic cross-sectional view of a sensing cell that is constructed by an NMOS and a suspended membrane. The gate of the NMOS is composed of the suspended membrane, and the source and drain regions are the two silicon diffusions under the membrane. The suspended membrane is a sandwiched structure including silicon dioxide, metal and silicon oxide layers, and each layer has a thickness of about 1 μm. The gap between the membrane and the substrate is about 0.65 μm. As shown in Figure 2, the dielectric of the NMOS is composed of a 1 μm oxide layer, a 0.65 μm air gap and a 1 μm oxide layer. The structure can be taken as a series of three capacitors and the total capacitance, *C_t_*, is given by:

(1) $$C_{t}=\frac{1}{\frac{1}{C_{ox}}+\frac{1}{C_{\text{gap}}}+\frac{1}{C_{ox}}}$$

where *C_ox_* and *C_gap_* represent the individual capacitance of the oxide and air gap, respectively [6]. The membrane produces a deformation upon applying a uniform pressure to the sensing cell, resulting in the total capacitance of the NMOS varies. In the saturation region, the drain current, *I_ds_*, of the NMOS is given by:

(2) $$I_{ds}=\frac{W\mu_{n}C_{t}}{2L}{\left(V_{gs}-V_{t}\right)}^{2}$$

where *W* and *L* represent the channel width and length of the NMOS, respectively; *μ_n_* is the mobility of the electrons; *V_t_* is the threshold voltage and *V_gs_* is the gate-to-source voltage [3]. In accordance with Equation (2), we know that the drain current, *I_ds_*, varies as the capacitance, *C_t_*, changes. Thereby, when applying a pressure to the membranes of the sensing cells, the pressure sensor generates a change in current.

**Figure 3.**
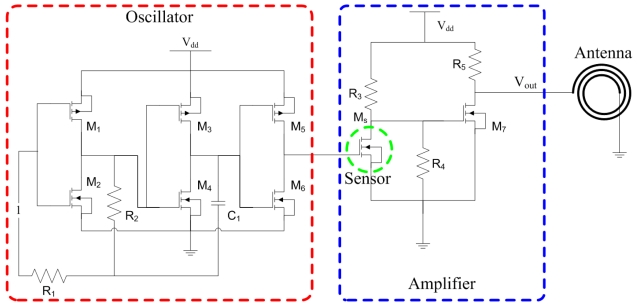
Circuits of the wireless pressure sensor.

Figure 3 illustrates the circuits of the wireless pressure sensor, where *M_1_, M_3_* and *M_5_* are PMOS; *M_2_, M_4_* and *M_6_* are NMOS. The oscillator is used to generate an ac signal providing to the gate of the FET pressure sensor.

Table 1 shows the channel length and width of all MOS in the wireless pressure sensor. The *M_1_, M_3_* and *M_5_* MOS have the same channel dimensions, and the channel width and length are 10 μm and 0.35 μm, respectively.

**Figure 4.**
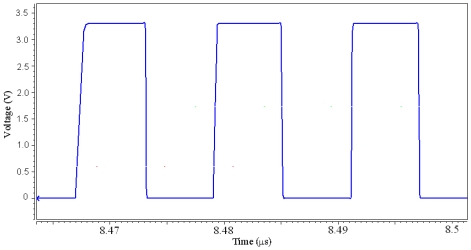
Output signal of the oscillator.

The *M_2_, M_4_* and *M_6_* MOS have the same channel dimensions, and these channels are 4 μm wide and 0.35 μm long. The resistance of *R_1_* and *R_2_* are 5 kΩ, and the capacitance of *C_1_* is 0.5 pF. We utilize the professional circuit simulation software, HSPICE, to simulate the output signal of the oscillator. Figure 4 displays the output signal of the oscillator. In this simulation, the input voltage *V_dd_* of 3.3 V is adopted. The simulated result shows that the oscillator can generate a square wave of 82 MHz.

**Figure 5.**
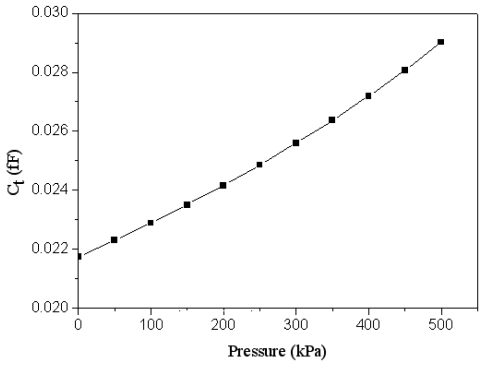
Relation of capacitance and pressure in the pressure sensor.

The finite element method software, CoventorWare, was employed to evaluate the relation of pressure and capacitance variation in the pressure sensor. A model of only one sensing cell was built since all sensing cells are the same. The triangular elements are adopted to mesh the model. The materials of the membrane are aluminum and silicon dioxide. The Young's modulus, Poisson's ratio and mass density of aluminum are 70 GPa, 0.3 and 2679 kg/m^3^, respectively, and the Young's modulus, Poisson's ratio and mass density of silicon dioxide are 69 GPa, 0.17 and 2200 kg/m^3^, respectively [13]. The load is a uniform pressure applied to the membrane, and the boundary condition is that the membrane edge is fixed. Figure 5 depicts the capacitance variation and pressure in the pressure sensor. The results show that the capacitance changes from 0.0218 to 0.029 fF as the pressure varies from 0 to 500 kPa.

**Figure 6.**
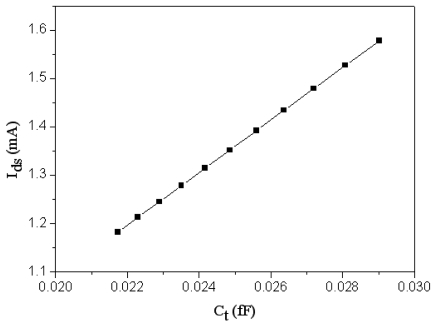
Relation of drain current and capacitance in the pressure sensor.

The channel width and length of the FET pressure sensor are 8 μm and 0.35 μm, respectively. Substituting the channel dimensions with *μ_n_* = 4.898 × 10^12^ m^2^/V·s, *V_t_* = 0.57 V and *V_gs_* = the output signal of the oscillator (Figure 4) into Equation (2), the relation of the drain current and the total capacitance in the pressure sensor can be yielded, and the result is plotted in Figure 6. The result depicts that the drain current varies from 1.18 to 1.6 mA as the capacitance changes from 0.218 to 0.029 fF.

**Figure 7.**
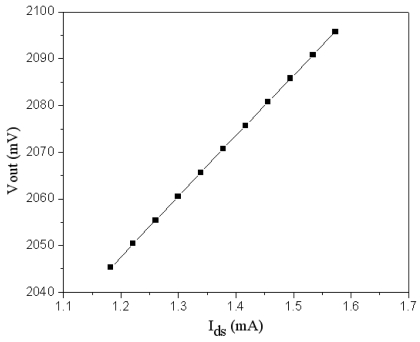
Output voltage of the amplifier.

As shown in Figure 3, the amplifier is utilized to convert the drain current of the FET pressure sensor into the voltage output. The channel width and length of *M_7_* MOS are 20 μm and 0.35 μm, respectively. The resistance of *R_3_, R_4_* and *R_5_* are 1 kΩ. We utilize HSPICE to simulate the output voltage of the amplifier. Figure 7 shows the simulated results of the amplifier, where the voltage *V_out_* is ac peak voltage. In this simulation, the input voltage *V_dd_* is 3.3 V. The results show that the output voltage of the amplifier increases from 2044 mV to 2095 mV when the drain current of the pressure sensor varies from 1.18 mA to 1.6 mA.

**Figure 8.**
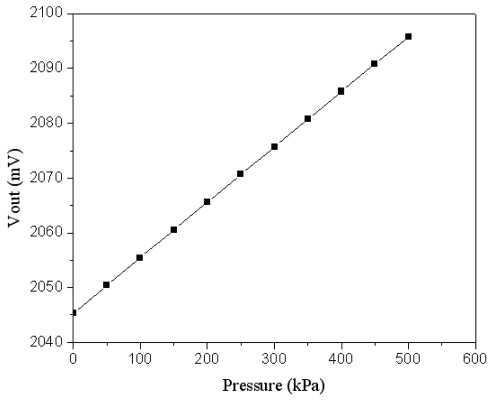
Simulated results of the output voltage in the pressure sensor.

Combining the results in Figures 5, 6 and 7, the output voltage of the pressure can be obtained. Figure 8 presents the relation of the output voltage and the pressure for the pressure sensor, where the voltage *V_out_* is ac peak voltage. The simulated results reveal that the output voltage of the pressure sensor changes from 2045 to 2095 mV while the pressure varies from 0 to 500 kPa.

## 3. Fabrication of Pressure Sensor

**Figure 9.**
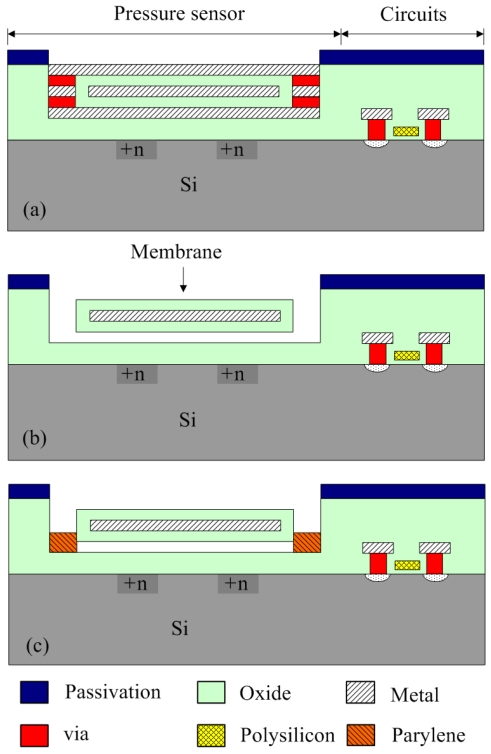
Process flow of the wireless pressure sensor: (a) after completion of CMOS process, (b) etching the sacrificial layers, and (c) sealing the etch holes.

The wireless pressure sensor was fabricated using the commercial 0.35 μm CMOS process of Taiwan Semiconductor Manufacturing Company (TSMC). Figure 9 illustrates the process flow of the wireless pressure sensor.

**Figure 10.**
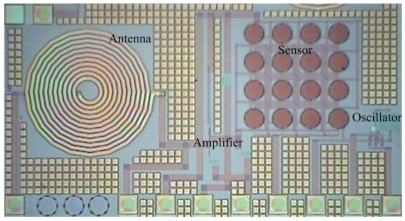
Photograph of the wireless pressure sensor after the wet etching process.

Figure 9a displays the schematic cross-section of the wireless pressure sensor after completion of the CMOS process. In the pressure sensor, the membranes are constructed by the oxide, metal and oxide layers. The sacrificial layers are the metal and via layers, which the materials of the metal and via layers are aluminum and tungsten, respectively. In order to obtain the suspended membranes, the pressure sensor requires a post-CMOS process [6] to remove the sacrificial layers. The post-process uses wet etching to etch the sacrificial layers, and to release the suspended membranes. Figure 9b shows that the pressure sensor is immersed in two etchants: one is an aluminum etchant with phosphoric acid, nitric acid, acetic acid and DI water in the ratio 14:1:2:3 and the other is a tungsten etchant with sulfuric acid and hydrogen peroxide in the ratio 2:1. The suspended membranes are released and an air gap between the membrane and substrate is formed after the sacrificial layers are removed. Figure 10 demonstrates the photograph of the wireless pressure sensor after the wet etching process.

**Figure 11.**
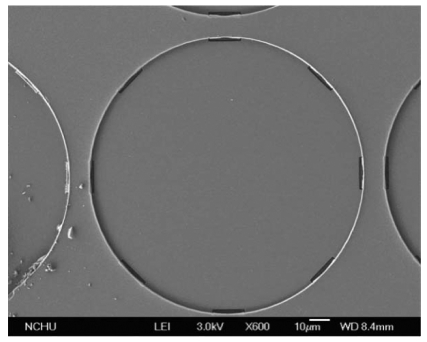
SEM image of a sensing cell after the wet etching process.

Figure 11 presents a scanning electron microscopy (SEM) image of a sensing cell after the wet etching process. The etch holes in the pressure sensor must be sealed. As shown in Figure 9(c), a LPCVD parylene is employed to seal the etch holes, and the parylene film is patterned by a dry etching. The thickness of the parylene film is about 2 μm. Because the LPCVD parylene is processed in a high vacuum chamber, the wireless pressure sensor that the cavities are nearly at vacuum is an absolute pressure sensor.

## 4. Results and Discussion

**Figure 12.**
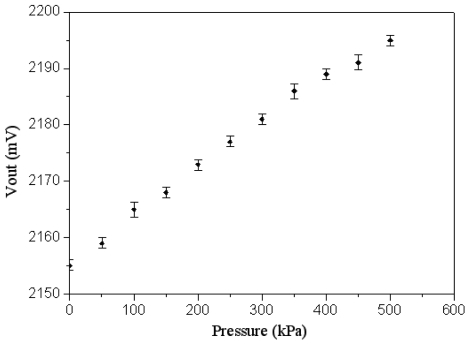
Measured results of the output voltage in the pressure sensor.

**Figure 13.**
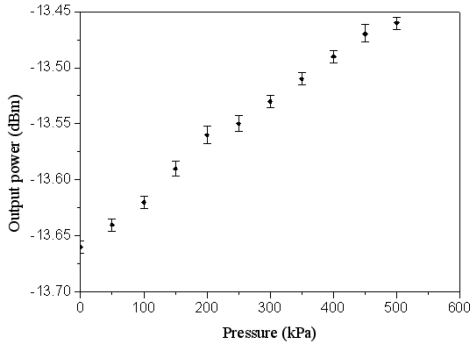
Output power of the pressure sensor.

The wireless pressure sensor was mounted in a pressure chamber. The nitrogen pressure source was provided to the pressure chamber, and the nitrogen pressure in the chamber could be tuned by the gas valves. A calibrated pressure sensor was employed to monitor the gas pressure in the pressure chamber. The power supply provided the *V_dd_* voltage of 3.3 V to the wireless pressure sensor. The output voltage of the pressure sensor was measured using an oscilloscope. Figure 12 shows the measured results of output voltage for the pressure sensor, where the voltage *V_out_* is ac peak voltage. In the measurement, the output voltage of the pressure sensor increased from 2155 to 2195 mV while the pressure varied from 0 to 500 kPa, so the sensitivity of the pressure sensor was about 0.08 mV/kPa. The measured results of output voltage in the pressure sensor were approximate to the simulated results in Figure 8. On the other hand, the output power of the pressure sensor was detected using a spectrum analyzer. Figure 13 displays the output power of the pressure sensor. The results depicted that the output power of the pressure sensor changed from -13.66 to -13.45 dBm as the pressure increased form 0 to 500 kPa.

**Figure 14.**
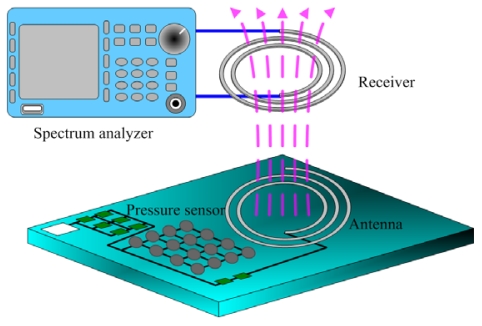
The output signal of the wireless pressure sensor received by a receiver.

In order to characterize the performance of transmission in the wireless pressure sensor, a receiver as shown in Figure 14 was used to receive the output signal of the antenna in the pressure sensor. The receiver, which was an inductor, used the magnetic coupling method to induce the transmitted signal of the antenna in the pressure sensor. The wireless pressure sensor and the receiver were set in the pressure chamber.

**Figure 15.**
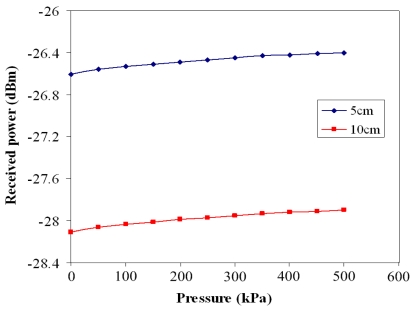
Received power of the receiver at the different transmission distances.

The *V_dd_* voltage of 3.3 V was supplied to the wireless pressure sensor, and the spectrum analyzer was adopted to measure the received power of the receiver. Figure 15 presents the received power of the receiver at different transmission distances.

At the transmission distance of 5 cm, the received power of the receiver was −26.606 dBm at zero pressure and −26.41 dBm at the pressure of 500 kPa. At the transmission distance of 10 cm, the receiver had a received power of −28.103 dBm at zero pressure and a received power of −27.91 dBm at the pressure of 500 kPa. Comparing the results in Figures 13 and 15, the output power of the wireless pressure sensor was −13.45 dBm at the pressure of 500 kPa, and the received power of the receiver was −26.41 dBm and −27.91 dBm at the transmission distance of 5 cm and 10 cm, respectively, under the pressure of 500 kPa. Thereby, the decay of power for the wireless transmission was −12.96 dBm at 5 cm distance and −14.46 dBm at 10 cm distance under 500 kPa pressure. On the other hand, the wireless pressure sensor chip had an input voltage of 3.3 V and an input current of 6.74 mA by the measurement, so the power consumption of the chip was about 22.24 mW.

An FET pressure sensor integrated with the readout circuit, reported by Dai *et al.* [6], was manufactured using the CMOS-MEMS technique, and the sensitivity of the pressure sensor was 0.032 mV/kPa. The pressure sensor in this work had a sensitivity of 0.08 mV/kPa. Therefore, the sensitivity of the pressure sensor in this work exceeded that of Dai *et al.* [6]. Furthermore, the pressure sensor in this study had the function of wireless transmission.

## 5. Conclusions

We have successfully fabricated a wireless pressure sensor using the commercial CMOS process and a post-process. The wireless micro pressure sensor was composed of a FET pressure sensor, an oscillator, an amplifier and an antenna. The oscillator produced an ac signal supplying to the gate of the pressure sensor. The sensing signal of the pressure sensor was amplified by the amplifier, and the output voltage of the pressure sensor was transmitted by the antenna. The FET pressure sensor required a post-process to release the suspended membranes after completion of the CMOS process. The post-process employed the etchants to etch the sacrificial layers to release the membranes of the pressure sensor, and then the etch holes in the pressure sensor were sealed by the LPCVD parylene. The fabrication of the wireless pressure sensor was compatible with the commercial CMOS process. The experiments revealed that the pressure sensor had a sensitivity of 0.08 mV/kPa in the 0–500 kPa pressure range and a wireless transmission distance of 10 cm.

## Figures and Tables

**Table 1.** Channel width and length of all MOS in the wireless pressure sensor.

**MOS**	**Width (μm)**	**Length (μm)**
M_1_	10	0.35
M_2_	4	0.35
M_3_	10	0.35
M_4_	4	0.35
M_5_	10	0.35
M_6_	4	0.35
M_7_	20	0.35
M_s_	8	0.35
